# Editorial: Diabetes and cardiovascular disease: new therapeutic interventions

**DOI:** 10.3389/fphar.2024.1472636

**Published:** 2024-08-12

**Authors:** Maria Consiglia Trotta, Michele D’Amico, Ludwig T. Weckbach, Anca Hermenean, Bartolo Ferraro

**Affiliations:** ^1^ Department of Experimental Medicine, University of Campania “Luigi Vanvitelli”, Naples, Italy; ^2^ German Center for Cardiovascular Research (DZHK), Partner Site Munich Heart Alliance, Munich, Germany; ^3^ Institute of Cardiovascular Physiology and Pathophysiology, Biomedical Center, Ludwig- Maximilian-University Munich, Munich, Germany; ^4^ Medizinische Klinik und Poliklinik I, Klinikum der Universität, Ludwig-Maximilians-University Munich, Munich, Germany; ^5^ Walter Brendel Centre of Experimental Medicine, Ludwig-Maximilians-University Munich, University Hospital, Munich, Germany; ^6^ Faculty of Medicine, Vasile Goldis Western University of Arad, Arad, Romania; ^7^ “Aurel Ardelean” Institute of Life Sciences, Vasile Goldis Western University of Arad, Arad, Romania

**Keywords:** diabetes, cardiovascular diseases, neuro-cardio-renal system, inflammation, therapies, drug discovery, experimental pharmacology

Diabetes mellitus (DM) is a chronic disease resulting from a deficiency in insulin production or the body’s inability to effectively use this hormone. The consequent chronic hyperglycemia leads to the development of retinopathy, nephropathy, neuropathy, and cardiovascular diseases (Cho et al., 2022).

The optimal control of blood glucose levels can be achieved by modifying lifestyle factors in combination with antidiabetic drugs such as biguanides, dipeptidyl peptidase 4 inhibitors (DPP-4i), sulfonylureas, meglitinides, thiazolidinediones (TZDs), sodium-glucose cotransporter inhibitors (SGLT2i), α-glucosidase inhibitors, glucose-dependent insulinotropic polypeptide (GIP) receptor, glucagon-like peptide-1 receptor agonists (GLP-1RAs) and the various types of insulin (Shahcheraghi et al., 2021). However, some of these medications may have important cardiovascular side effects (Alvarez et al., 2015). Therefore, this Research Topic aimed to generate new therapeutic interventions capable of counteracting the negative effects of diabetes on the cardiovascular system.

In a multicenter observational study, Sardu et al. enrolled 334 diabetic patients with left bundle branch (LBB) block receiving LBB pacing for cardiac resynchronization therapy (CRT). The rate of CRT responders to LBB pacing was evaluated at 1-year follow-up, along with the causes of death, cardiac death, heart failure (HF) hospitalization events, and the expression of selected microRNAs. At 1-year follow-up, patients who responded to LBB pacing showed increased expression of several microRNAs, including miR-26, miR-29, miR-30, miR-92, and miR-145. Increased miR-30 expression was associated with significant improvement of cardiac function in patients with type 2 diabetes mellitus (T2DM), through a reversion of left ventricular remodelling. This would pave the way to test the effects of specific treatment with mimic-miR on cardiac remodeling in patients treated with LBB pacing.

A network meta-analysis by Huang et al. reveals, for the first time, that different gliflozins have different impacts on various cardiovascular and respiratory outcomes. The study includes twenty-nine randomized controlled trials involving 100,740 subjects. The results of such meta-analysis show that sotagliflozin significantly reduced myocardial infarction, cardiac failure, chronic and congestive cardiac failure, complete atrioventricular block, and pneumonia; while empagliflozin reduced acute cardiac failure, acute respiratory failure, and hypertensive crisis. In addition, dapagliflozin reduced asthma and hypertensive emergency while canagliflozin significantly reduced respiratory tract infections.

An interesting comparison between SGLT2i and DPP4i effects on cardiovascular outcomes in diabetic patients has been performed by Chen et al. The analysis evidenced that a positive autonomic modulation emerged in patients treated with SGLT2i, showing also favorable cardiovascular and renal outcomes. On the contrary, detrimental effects on neuroelectrocardiography were evident in patients treated with DDP4i. Based on these data, SGLT2i could be recommended not only as a first/second-line medication in T2DM patients but also as an effective treatment for HF, independently from DM.

From a different perspective, Riemma et al. extensively described the beneficial effects of SGLT2i and GLP-1RAs in heart failure and cognitive impairment. The authors evidenced that, beyond their efficacy in gaining glycemic control in diabetic patients, both SGLT2i and GLP-1RAs exert cardioprotective and neuroprotective actions through the modulation of oxidative stress, inflammation, insulin signaling, and overload of ions. Therefore, the use of anti-diabetic drugs could be suggested to improve cardiometabolic profile and cognitive impairment in diabetic patients, independently from their anti-diabetic action.

The review by Fu et al. considered recent large-scale clinical trials and basic research articles on the use of SGLT2i, GLP-1RAs, and DPP-4i. It highlighted their cardiorenal protective effects, including the involvement of glucose-dependent and independent pathways, and underscored their clinical implications beyond glycemic management.

Xie et al. compared the effects of eplerenone, an aldosterone receptor antagonist, administered alone or in combination with ADAM17 knockdown in C57BL/6J mice receiving intraperitoneal streptozotocin (STZ) to mimic diabetic cardiomyopathy (DCM). The combined treatment significantly reduced cardiac hypertrophy, fibrosis, and dysfunction. Reduction of transforming growth factor beta 1 (TGF-β1)/Smad3 pathway was observed in both STZ-mice and cardiac fibroblasts exposed to high glucose levels. Therefore, the combination of eplerenone and ADAM17 knockdown could represent a potential therapeutic strategy in DCM, which requires further clinical investigations.

A different novel approach aimed at attenuating cardiac fibrosis induced by chronic diabetes was proposed by Trotta et al., through the formulation of a new drug delivery system (DDS) combining two anti-fibrotic molecules, chrysin (CHR) and the calixarene OTX008 (a Galectin-1 inhibitor) with sulfobutylated β-cyclodextrin (SBECD). The new DDS was tested in hyperglycemic H9c2 cardiomyocytes and STZ-CD1 mice. The treatment notably improved H9c2 cell morphology and viability. Moreover, it reduced Galectin-1 (a pro-fibrotic mediator) and TGF/Smad pathway both *in vitro* and in hearts from mice with chronic diabetes, showing an improved cardiac remodeling and extracellular matrix (ECM) composition. Overall, the novel DDS was able to increase CHR and OTX solubility/bioavailability *in vivo* and to counteract the cardiac fibrosis induced by prolonged hyperglycemia.

Through the prediction of pharmacokinetics, network pharmacology, and molecular docking, the study by Huang et al. identified the combination of rosmarinic acid, luteolin, and resveratrol as potent anti-diabetic molecules. Both *in vitro* α-Amylase inhibition assay and *in vivo* tests, such as oral starch tolerance test and oral glucose tolerance test in diet-induced obese diabetic mice, have demonstrated the predicted hypoglycemic effect of this mixture, capable of addressing the issue of low availability and enhance the efficacy of the single compounds. These promising results could have important implications for human health, although they need to be validated in clinical trials.

In another interesting study, An et al. investigated the impact of celastrol, a pentacyclic triterpene, on hyperglycemia-induced endothelial dysfunction. Low doses of celastrol were effective in attenuating superoxide and pro-inflammatory cytokines production in HUVEC cells exposed to high glucose and palmitic acid-containing media, by increasing the activity of nuclear factor (erythroid-derived 2)-like protein 2 (Nrf2). Furthermore, diabetic db/db mice treated with celastrol showed reduced blood glucose concentration and improved insulin sensitivity after fasting. The same mouse strain showed decreased capillary density in a wound healing model when injected intravenously with an adeno-associated virus (AAV9) harboring Nrf2 short hairpin (sh)RNA. Taken together, these findings may point at celastrol as a future new therapeutic approach for the short-term treatment of refractory diabetes-related skin ulcers and vascular defects.

The review by Zhang et al. focused on the importance of ferroptosis, a new form of cell death characterized by iron-dependent lipid peroxidation, in the pathogenesis of diabetic cardiovascular diseases (CVDs). The manuscript also summarizes the positive effects of traditional Chinese medicine in CVD prevention, by focusing on the regulation of ferroptosis by polyphenols, alkaloids, and saponins. This evidence provides a new scientific basis for innovative management of CVDs through modulation of ferroptosis.

Collectively, these studies tackle diabetes-induced cardiovascular complications from different perspectives and provide exciting new therapeutic approaches for their management.
